# Supplementary material for: Diurnal dynamics and psychobiological regulation of cell-free mitochondrial and nuclear DNA in human saliva
Source: medRxiv. 2025 Oct 15:2025.10.13.25337887. Preprint. [Version 1] doi: 10.1101/2025.10.13.25337887 (PMC12633117; doi:10.1101/2025.10.13.25337887)
Supplement: Supplement 1 [file media-1.pdf]

Supplementary Materials for:

Diurnal dynamics and psychobiological regulation of cell-free mitochondrial and nuclear DNA in human saliva

Alexander Behnke, PhD*, David Shire, PhD, Samantha Leonard, BS, Aidan Park, BS, Lauren Petri, BS, Anjali Goyal, BS, Clemens Kirschbaum, PhD, Martin Picard, PhD, Caroline Trumpff, PhD*

* Corresponding Authors' Contact Information:

Alexander Behnke, PhD
Universität Ulm
Albert-Einstein-Allee 47
D-89081 Ulm, Germany
Tel.: +49 731 5026597
Email: alexander.behnke@uni-ulm.de

Caroline Trumpff, PhD
Columbia University Irving Medical Center
622 West 168th St., PH 1540O
New York, NY 10032
Tel: +1 703 8701609
cat2184@cumc.columbia.edu

Supplementary Methods

Inclusion and exclusion criteria

Inclusion Criteria:

- Healthy male and female adults aged 20−50 years.
- Ability and willingness to provide saliva samples, wear actigraphy and heart rate monitors, and complete questionnaires.
- Capacity to provide informed consent and participate in the study.
- For female participants, use of an effective birth control method.
- Body Mass Index (BMI) between 18.5 and 29.9, calculated from self-reported height and weight.
- Fluency in English.
- Regular menstrual cycles (21−35 days) for premenopausal women, specifically during the luteal phase (days 21−28 of the cycle).
- Access to a freezer for saliva sample storage.

Exclusion Criteria:

- History or current diagnosis of oral health issues (e.g., periodontitis, gingivitis).
- Diagnosis of early menopause (absence of menses for ≥2 months).
- Employment as a shift worker.
- Diagnosis of sleep disorders, including insomnia.
- Current pregnancy or potential pregnancy.
- Active cancer or chronic inflammatory disorders.
- Use of medications affecting cardiovascular or inflammatory systems (e.g., NSAIDs, corticosteroids, β-blockers).
- Presence of cold or flu symptoms within the last four weeks.
- Current use of nicotine or tobacco products.

Cell-free mitochondrial DNA quantification

Mitochondrial and nuclear DNA in cell-free saliva were quantified using a previously established method [1] with the following changes and additional details. In instances where the cited method indicates that plates are to be vortexed, they were instead shaken at 1,500 rpm for 1 minute on an OrbiShaker MP (Benchmark Scientific, Sayreville, NJ) and then briefly centrifuged using an Axygen plate centrifuge (Corning Life Sciences, Corning, NY). Models of thermal cyclers used for lysis were either T100 (Bio-Rad, Hercules, CA) or ProFlex PCR System (Applied Biosystems, Waltham, MA). Instead of preparing standard curves using DNA extracted from human fibroblasts (hFB), they were prepared using a chimeric ND1-B2M synthetic DNA fragment (sND1-B2M; gBlocks® gene fragment, Integrated DNA Technologies, Coralville, IA). Both

templates are described in the published method [1]. The absolute copy numbers of target genes in sND1-B2M were determined by quantifying it against hFB, which had been previously quantified by digital droplet polymerase chain reaction (Figure SX1). Instead of using the averages of triplicate C_T measurements after discarding outliers for quantification, the medians of triplicate C_T measurements (C_{Tmed}) without outlier removal were used. For each qPCR plate, linear regressions were fit to C_{Tmed} versus natural log-transformed (Ln) copy number for each target gene in the serially diluted standard. Absolute copy numbers of target genes in samples were calculated using the following formula:

$$Copy\ per\ reaction = e^{\frac{C_{Tmed}-b}{m}}$$

Where b and m are the slope and intercept of the linear regression of the standard curve for the corresponding qPCR plate and target gene. A target gene was considered below the limit of detection (LOD) when its copy number per reaction was three or fewer [2]. Copy per reaction values were transformed to copy per µL saliva by correcting for dilution of samples into lysis and qPCR buffers. Eight reference standards, created by pooling samples from the Picard lab biobank, were loaded on each 96-well plate alongside saliva samples prior to lysis to assess and correct for plate effects. The measurements of these standards were used to calculate correction factors that were specific to each pair of replicate plates as follows:

$$cf_p = \frac{\overline{LN(ND1)}_p}{\overline{LN(ND1)}_{exp}}$$

Where cf_p is the correction factor for plate p, and $\overline{LN(ND1)}_p$ and $\overline{LN(ND1)}_{exp}$ are the averages of LN ND1 copy number per µL in reference standards on plate p and experiment-wide, respectively. All measurements of ND1 and B2M in samples were then adjusted by dividing their LN values by the correction factor for their corresponding plate. Adjusted values were used in subsequent statistical analyses.

Diurnal Biomarker Metrics

We computed five different metrics to capture complementary aspects of the shape, variability, and regulatory characteristics of the diurnal biomarker profile. For the calculation, we used the following formula, with y_i indicating the values of respective biomarker at time t_i indicating the effective sampling time point, ranging from Awakening (i = 1) to a maximum of i_{max} = 20 samplings per day:

(a) Awakening level: S1 = $\ln(y_1)$

(b) Awakening response: AR = $\frac{y_{peak}}{y_1}$, using the concentrations at the diurnal peak (y_{peak}) located at t = 180 min for cell-free DNAs and at t = 30 to 45 min for cortisol.

(c) Daily average: AV = $Mean(\ln(y_i))$, if at least 60% of the samplings per day were available and timed

(d) Diurnal variability: %CV = $\frac{SD(\ln(y_i))}{Mean(\ln(y_i))} \cdot 100\%$

(e) Three-point diurnal AUC above baseline: AUCi = $\sum_{i=1}^{n-1}\left(\frac{y_i+y_{i+1}}{2} \cdot (t_{i+1} - t_1)\right) - (t_n - t_1) \cdot y_1$, where y_i are the $\log_e$-scaled values of respective biomarker at the sampling time points t_i, with t_1 = Awakening, t_2 = Peak, t_3 = Bedtime. Computation is only done if all n = 3 time points are available. Note that t_2 was +180 min post-awakening for cell-free DNAs and the maximum value at either +30 or +45 min post-awakening for cortisol.

Note that simplified estimations were used, for example, for the awakening response, as the amount of missing and not-timed samplings did not allow to compute areas under the curve integrating all samplings from awakening to the marker's diurnal peak [3].

Supplementary Tables

Supplementary Table S1. Detection and missing frequencies of salivary biomarkers among all available samples (*n* = 828)

	Quantifiably detected [a]	Semi-quantifiably detected [b]	Not detected [c]	Not measured
Cf-mtDNA	826 (99.8%)	–	–	2 (0.2%)
Cf-nDNA	715 (86.4%)	–	111 (13.4%)	2 (0.2%)
Cortisol	306 (37.0%)	–	–	520 (62.8%)[d]
Cortisone	306 (37.0%)	–	–	520 (62.8%)[d]
Corticosterone	96 (11.6%)	–	210 (25.4%)	520 (62.8%)[d]
DHEA	204 (24.6%)	–	102 (12.3%)	520 (62.8%)[d]
Testosterone	262 (31.6%)	44 (5.3%)	–	520 (62.8%)[d]
Progesterone	306 (37.0%)	–	–	520 (62.8%)[d]
Estradiol	154 (18.6%)	108 (13.0%)	44 (5.3%)	520 (62.8%)[d]

Note: Absolute frequencies and percentages relative to the total number of available saliva samples, *n* = 828, collected from 25 participants over two days, with a maximum of 20 samples per day. [a] Concentrations were above the limit of quantification (LOQ). [b] Concentrations were below the limit of quantification (LOQ) and above the limit of detection (LOD). [c] Concentrations not detectable or below the LOD were replaced with the LOD/√2. [d] The measurement of cell-free DNA was prioritized over hormone measurements in terms of available material and financial resources, ultimately resulting in 306 saliva samples being used for hormone measurements.

Supplementary Table S2. Detection and missing frequencies of salivary biomarkers among timed samples (n = 576)

	Quantifiably detected [a]	Semi-quantifiably detected [b]	Not detected [c]	Not measured
Cf-mtDNA	574 (99.7%)	−	−	2 (0.3%)
Cf-nDNA	489 (84.9%)	−	85 (14.8%)	2 (0.3%)
Cortisol	247 (42.9%)	−	−	329 (57.1%)
Cortisone	247 (42.9%)	−	−	329 (57.1%)
Corticosterone	83 (14.4%)	−	164 (28.5%)	329 (57.1%)
DHEA	168 (29.2%)	−	79 (13.7%)	329 (57.1%)
Testosterone	219 (38.0%)	28 (4.9%)	−	329 (57.1%)
Progesterone	247 (42.9%)	−	−	329 (57.1%)
Estradiol	120 (20.8%)	87 (15.1%)	40 (6.9%)	329 (57.1%)

Note: Absolute frequencies and percentages relative to the number of timely collected saliva samples, n = 576, collected from 25 participants over two days, with a maximum of 20 samples per day. The measurement of cell-free DNA was prioritized over hormone measurements in terms of available material and financial resources, ultimately resulting in 306 saliva samples being used for hormone measurements, of which 247 were collected timely. [a] Concentrations were above the limit of quantification (LOQ). [b] Concentrations were below the limit of quantification (LOQ) and above the limit of detection (LOD). [c] Concentrations not detectable or below the LOD were replaced with the LOD/√2.

Supplementary Table S3. Results of model comparisons for the relevance of sampling day

Outcome	Fixed terms	Random terms	AICc	BIC	R^2_{marg}	R^2_{cond}
Cf-mtDNA	Time	ID + ID:Day	**12944.9**	**13043.0**	**.078**	**.588**
	Time × Day	ID + ID:Day	12970.5	13150.5	.090	.596
Cf-nDNA	Time	ID + ID:Day	**8458.3**	**8583.5**	**.118**	**.590**
	Time × Day	ID + ID:Day	8515.4	8695.4	.128	.594
DNA ratio	Time	ID + ID:Day	**6105.9**	**6204.0**	**.080**	**.475**
	Time × Day	ID + ID:Day	6125.3	6305.4	.107	.480
Cortisol	Time	ID + ID:Day	**533.4**	**609.1**	**.585**	**.734**
	Time × Day	ID + ID:Day	566.2	698.5	.593	.736
Cortisone	Time	ID + ID:Day	**1305.6**	**1381.4**	**.555**	**.675**
	Time × Day	ID + ID:Day	1336.4	1468.7	.565	.681
Testosterone	Sex × Time	ID + ID:Day	**1844.5**	**1974.2**	**.874**	**.941**
	… + Time × Day	ID + ID:Day	1879.8	2054.9	.872	.943
Progesterone	SexCycle × Time	ID + ID:Day	**915.0**	**1082.1**	**.162**	**.742**
	… + Time × Day	ID + ID:Day	971.5	1171.8	.175	.742
DHEA	Time	ID:Day[a]	**2851.6**	**2924.3**	**.148**	**.181**
	Time × Day	ID:Day[a]	2884.9	3014.6	.193	.229
Estradiol	Sex × Time	ID:Day[a]	**1357.5**	**1484.6**	**.142**	**.162**
	… + Time × Day	ID:Day[a]	1375.1	1548.3	.250	.310

Note. Linear mixed-effects models with transformed outcome scales using the natural logarithm to adjust excessive right skew, except for DNA ratio. We modelled a repeated data structure using random intercepts for subjects and sampling day (weekday vs. weekend) nested within subjects. To check whether time trends systematically differ between weekdays and weekend, we considered the sampling day as fixed effect. Model fit indices (AICc, BIC) suggest that sampling day does not improve the model fit when considered as a fixed effect. Thus, all main results are based on models specifying the sampling day as a random intercept only. Models mapping the trajectories of hormones with sex effects and/or effects of the menstrual cycle specified either assigned sex (here, concordant with gender identity) or a combined sex/phase variable (male vs. female-follicular vs. female-luteal) as null model. [a] Random intercept for subjects (ID) was singular and has been removed.

Supplementary Table S4. Results of linear mixed-effects models of diurnal biomarker trajectories

**Outcome**	**Model terms**	**Test statistics**			**Effect size**
Cf-mtDNA	*Fixed effects*	***F***	***df*s**	***p***	η^2_{part}
	Intercept	2009.84	1, 24.04	< .001	
	Time	5.28	19, 513.62	< .001	.16
	Random effects	**LR-χ²**	***df***	***p***	σ_{random}
	ID	9.77	1	.002	0.93
	ID:Day	50.68	1	< .001	0.67
	Overall model	***F***	***df*s**	***p***	R^2_{marg} (R^2_{cond})
		5.28	19, 513.62	< .001	.078 (.588)
Cf-nDNA	*Fixed effects*	***F***	***df*s**	***p***	η^2_{part}
	Intercept	503.19	1, 24.04	< .001	
	Time	7.89	19, 514.04	< .001	.23
	Random effects	**LR-χ²**	***df***	***p***	σ_{random}
	ID	10.62	1	.001	1.09
	ID:Day	56.34	1	< .001	0.76
	Overall model	***F***	***df*s**	***p***	R^2_{marg} (R^2_{cond})
		7.89	19, 514.04	< .001	.118 (.590)
DNA ratio	*Fixed effects*	***F***	***df*s**	***p***	η^2_{part}
	Intercept	870.29	1, 24.03	< .001	
	Time	4.29	19, 518.74	< .001	.14
	Random effects	**LR-χ²**	***df***	***p***	σ_{random}
	ID[a]	21.31	1	< .001	0.63
	ID:Day	6.75	1	.009	0.23
	Overall model	***F***	***df*s**	***p***	R^2_{marg} (R^2_{cond})
		4.29	19, 518.74	< .001	.080 (.475)
Cortisol	*Fixed effects*	***F***	***df*s**	***p***	η^2_{part}
	Intercept	3.09	1, 18.15	.096	
	Time	24.20	19, 205.85	< .001	.69
	Random effects	**LR-χ²**	***df***	***p***	σ_{random}
	ID	6.91	1	.009	0.41
	ID:Day	1.14	1	.284	0.14
	Overall model	***F***	***df*s**	***p***	R^2_{marg} (R^2_{cond})
		24.20	19, 205.85	< .001	.585 (.734)
Cortisone	*Fixed effects*	***F***	***df*s**	***p***	η^2_{part}
	Intercept	470.47	1, 18.10	< .001	
	Time	19.34	19, 206.15	< .001	.64

	Random effects	**LR-χ^2**	***df***	***p***	**σ_{random}**
	ID	3.63	1	.057	0.24
	ID:Day	2.49	1	.114	0.14
	Overall model	***F***	***df*s**	***p***	**R^2_{marg} (R^2_{cond})**
		19.34	19, 206.15	< .001	.555 (.675)
Testosterone	*Fixed effects*	***F***	***df*s**	***p***	**η^2_{part}**
	Intercept	605.07	1, 121.31	< .001	
	Sex	49.49	1, 121.56	< .001	.29
	Time	3.22	19, 184.12	< .001	.25
	Time × Sex	1.83	18, 184.19	.024	.15
	Random effects	**LR-χ^2**	***df***	***p***	**σ_{random}**
	ID	8.09	1	.004	0.24
	ID:Day	11.26	1	< .001	0.14
	Overall model	***F***	***df*s**	***p***	**R^2_{marg} (R^2_{cond})**
		9.09	38, 173.89	< .001	.874 (.941)
Progesterone	*Fixed effects*	***F***	***df*s**	***p***	**η^2_{part}**
	Intercept	413.21	1, 91.96	< .001	
	SexCycle	0.28	2, 99.03	.754	.00
	Time	1.26	19, 171.58	.217	.12
	Time × SexCycle	1.30	33, 169.88	.146	.20
	Random effects	**LR-χ^2**	***df***	***p***	**σ_{random}**
	ID	25.95	1	< .001	0.21
	ID:Day	0.76	1	.382	0.03
	Overall model	***F***	***df*s**	***p***	**R^2_{marg} (R^2_{cond})**
		1.09	54, 157.91	.343	.162 (.742)
DHEA	*Fixed effects*	***F***	***df*s**	***p***	**η^2_{part}**
	Intercept	1049.97	1, 19.90	< .001	
	Time	2.18	19, 214.04	.004	.16
	Random effects	**LR-χ^2**	***df***	***p***	**σ_{random}**
	ID[a]	–	–	–	–
	ID:Day	1.19	1	.275	0.27
	Overall model	***F***	***df*s**	***p***	**R^2_{marg} (R^2_{cond})**
		2.18	19, 214.04	.004	.148 (.181)
Estradiol	*Fixed effects*	***F***	***df*s**	***p***	**η^2_{part}**
	Intercept	11.41	1, 207.92	< .001	
	Sex	0.00	1, 208.00	.998	.00
	Time	0.89	19, 197.43	.598	.08
	Time × Sex	1.11	18, 197.50	.348	.09

Random effects	LR-χ^2	*df*	*p*	σ_{random}
ID[a]	–	–	–	–
ID:Day	0.35	1	.555	0.14
Overall model	***F***	***df*s**	***p***	R^2_{marg} (R^2_{cond})
	1.07	38, 186.69	.373	.142 (.162)

Note. Outcomes were transformed using the natural logarithm to adjust excessive right skew. Models mapping the trajectories of hormones with sex effects and/or effects of the menstrual cycle specified either assigned sex (here, concordant with gender identity) or a combined sex/phase variable (male vs. female-follicular vs. female-luteal). [a] Random intercept for subjects (ID) was singular and has been removed. Analyses of cell-free mitochondrial DNA (cf-mtDNA) and cell-free nuclear DNA (cf-nDNA) and their ratio based on n_{obs} = 574 observations in 49 trajectories from N = 25 participants; cortisol, cortisone, testosterone, progesterone, DHEA, and estradiol models based on n_{obs} = 247 observations in 33 trajectories from N = 17 participants. Post hoc tests for significant effects are provided in the Supplementary Data Table D1.

Supplementary Table S5. Spearman correlations between cell-free DNA and hormone levels in saliva in contemporaneous samples (n = 304)

	Cf-mtDNA		***Cf-nDNA***	
	r_S	p	r_S	p
Cortisol	−.26	< .001	−.34	< .001
Cortisone	−.25	< .001	−.32	< .001
Corticosterone	−.26	< .001	−.21	< .001
DHEA	−.06	.284	−.10	.087
Testosterone	.27	< .001	.07	.242
Progesterone	−.15	.008	.01	.858
Estradiol	−.13	.027	−.09	.135

Supplementary References

1. Michelson J, Rausser S, Peng A, Yu T, Sturm G, Trumpff C *et al.* MitoQuicLy: A high-throughput method for quantifying cell-free DNA from human plasma, serum, and saliva. *Mitochondrion* 2023; **71**: 26–39.

2. Bustin SA, Benes V, Garson JA, Hellemans J, Huggett J, Kubista M *et al.* The MIQE Guidelines: Minimum Information for Publication of Quantitative Real-Time PCR Experiments. *Clin Chem* 2009; **55**: 611–622.

3. Hellhammer J, Fries E, Schweisthal OW, Schlotz W, Stone AA, Hagemann D. Several daily measurements are necessary to reliably assess the cortisol rise after awakening: State- and trait components. *Psychoneuroendocrinology* 2007; **32**: 80–86.
